# Key questions on the epigenetics of herpes simplex virus latency

**DOI:** 10.1371/journal.ppat.1010587

**Published:** 2022-06-30

**Authors:** Abigail L. Whitford, Anna R. Cliffe

**Affiliations:** Department of Microbiology, Immunology and Cancer Biology, University of Virginia, Charlottesville, Virginia, United States of America; University of Arizona, UNITED STATES

The human pathogen, herpes simplex virus (HSV), has established a successful mechanism to persist long term in its host by maintaining a latent infection in neurons. Latent infection is defined as long-term carriage of the viral genome but lack of detectible infectious virus and the ability to reactivate following a specific stimulus. During latency, the HSV genome exists as a nuclear, unintegrated episome. Although multiple factors regulate entry into latent infection, the epigenetic structure of the latent HSV genome in neurons differs from the configuration of the viral genome during lytic infection of nonneuronal cells and therefore appears to play a central role in regulating viral gene transcription. Since the initial discoveries of chromatinization of the HSV genome during latency, multiple new developments have been made in how the viral genome could be epigenetically silenced in a way that promotes long-term persistence while maintaining the ability to reactivate. Here, we highlight recent developments and incorporate new questions that will ultimately help understand how HSV latency occurs in neurons and how the epigenetic structure of the viral genome may ultimately impact clinical HSV-associated disease.

## What is the epigenetic environment of the latent HSV genome?

Heritable alterations in gene expression that are not encoded by the DNA sequence are known as epigenetic modifications. Gene silencing of cellular, nonviral DNA can be mediated by DNA methylation. Thus far, there is limited evidence of canonical CpG methylation on the HSV genome [1]. This is based on a lack of canonical CpG methylation on regions of the viral genome containing high levels of CpG such as the latency-associated transcript (LAT) region and the viral lytic gene, ICP4 [1]. However, the host genome in neurons is subject to a high degree of non-CpG methylation, which is laid down robustly during the critical period of neuronal maturation (in humans up to 16 years, in mice up to 4 weeks) [2]. It remains to be determined whether the latent HSV genome is subject to non-CpG methylation and whether the levels are altered depending on the timing of neuronal infection.

**Fig 1:**
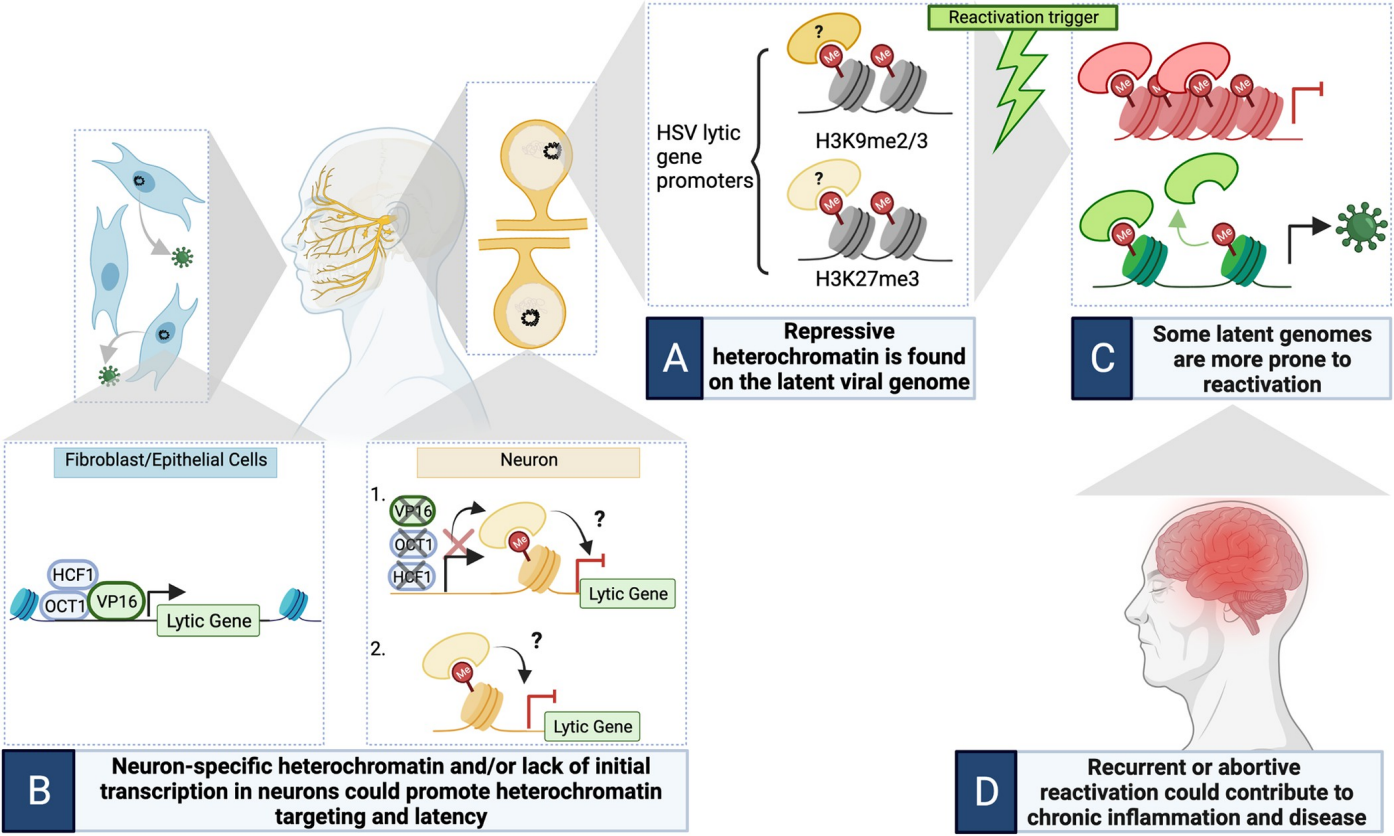
The epigenetics of HSV latency. **(A)** Repressive heterochromatin is found on the latent viral genome. The repressive heterochromatin marks H3K9me2/3 and H3K27me3 are both known to be enriched on the latent viral genome and have a restrictive effect on the viral DNA. What histone readers associate with these marks and the effects of various histone readers on viral DNA is unknown. **(B)** Neuron-specific heterochromatin and/or lack of initial transcription in neurons could promote heterochromatin targeting and latency. (B1) Heterochromatin could form on the HSV genome secondary to reduced viral transcription to maintain silencing. (B2) Neuron-specific heterochromatin types may directly repress HSV gene expression. The mechanisms of heterochromatin targeting to the HSV genome in neurons are not understood. **(C)** Some latent genomes are more prone to reactivation. After a reactivation stimulus, only a subpopulation of latent genomes will reactivate. The differences in histone PTMs, histone associated proteins, and overall 3D compaction of the viral genome could contribute to the heterogeneity of reactivation. **(D)** Recurrent or abortive reactivation from HSV genomes in an epigenetic state more prone to reactivation could contribute to chronic inflammation and disease. HSV infection potentially contributes to neurological disease, such as late onset Alzheimer disease, potentially via recurrent reactivation, leaky latency, and prolonged inflammation. Figure created with BioRender.com. HSV, herpes simplex virus; PTM, posttranslational modification.

Cellular DNA is further condensed when wrapped around an octamer of histone proteins, H2A, H2B, H3, and H4, to form a nucleosome. Micrococcal nuclease digestion of viral DNA in the brainstem of mice indicates a nucleosomal structure is present on the viral genome [3]. Histone H3 is associated with the HSV genome during latency and its amino terminus can be subject to posttranslational modifications (PTMs) that affect gene transcription. During HSV latency, marks associated with repressive heterochromatin, including histone di- and tri-methyl lysine 9 (H3K9me3) and histone trimethyl lysine 27 (H3K27me3), are abundant on transcriptionally inactive areas, such as lytic promoters (Fig 1A) [4–7]. In contrast, a region of the viral genome containing the promoter and enhancer LAT, which undergoes robust transcription during latency, is enriched for euchromatin marks including H3K4me3 as well as acetylation on histone H3 [1,4].

Although histone PTMs can directly regulate gene expression, the major epigenetic consequences result from binding of histone reader proteins. Methylated H3K9 can be read by heterochromatin proteins 1 (HP1) and ATRX on cellular chromatin [8], but it is unknown if these proteins act as readers on viral DNA. H3K27me3 is read by Polycomb group repressive complexes (PRCs). The complex that adds the H3K27me3 mark, and also has H3K27me3 reading function (PRC2), is known to associate with the HSV genome following the resolution of acute infection in mice [9]. A second complex, known as PRC1, exists in multiple forms and can bind H3K27me3 to mediate gene silencing via genome compaction, three-dimensional interactions, and/or formation of phase separated domains [10]. A component of PRC1, Bmi1, has only limited association with the latent HSV genome [6,9]. Therefore, it is likely that PRC1 has either limited association or different forms of PRC1 lacking Bmi1 associate with the latent viral genome. The viral genome is thus epigenetically regulated, but there are still uncertainties as to what other histones, histone modifications, and histone readers are enriched on the viral genome, as well as whether different histone PTMs are located on the same or distinct populations of HSV genomes.

## How do neuronal epigenetics contribute to the initiation and maintenance of HSV gene silencing?

HSV only establishes a latent infection in postmitotic neurons. Thus, understanding what makes neurons epigenetically unique to other cell types could have significant implications to our understanding of latency. Although it is unclear why certain histone PTMs become enriched during latency and how these changes contribute to the onset and maintenance of latent infection, one possibility is that certain forms of epigenetic silencing occur secondarily to a reduction in lytic gene transcription. For example, the timing of H3K27me3 formation on the latent genome suggests that this modification is more important in maintenance, but potentially not the initiation, of gene silencing [9]. The complex responsible for formation of H3K27me3 can be targeted indiscriminatingly to regions of chromatin but can only methylate H3K27 in the absence of active transcription and euchromatic modifications [11]. Neuronal-specific microRNAs have been shown to limit the levels of transcription factors (Oct1 and FOXC1) that promote HSV lytic gene transcription and limit heterochromatin formation [12]. In particular, Oct1 forms a complex with the viral transactivator VP16 and HCF1, both of which display distinct localization patterns in neurons. HCF1 displays a cytoplasmic localization in sensory neurons [13] and VP16 trafficking along axons is slower than nucleocapsid trafficking, resulting in a reduced ability to transactivate lytic genes following neuronal axon-specific infections [14,15]. As a result, due to a lack of transcription factors, some forms of epigenetic modifications could be secondary to reduced transcription in neurons (Fig 1B, panel 1).

Neurons are a specialized, terminally differentiated, postmitotic cell type with unique patterns of gene expression and response to stimuli. Therefore, it is also plausible that the unique chromatin environment and resulting differential expression or function of host proteins or RNA in neurons contributes to epigenetic silencing in HSV latency to actively drive gene repression (Fig 1B, panel 2). The composition of the Polycomb Group complexes changes following neuronal differentiation and during maturation. This includes a switch in the predominant H3K27 methyltransferase as EZH1 replaces EZH2 as the major species present in mature neurons [16]. In addition, the relative levels of PRC1 proteins also changes with neuronal maturation [17]. Polycomb group complexes display considerable heterogeneity in their protein composition, which can result in different consequences on chromatin structure [10]. Therefore, it is probable that are multiple unexplored differences in the epigenetic environment of neurons compared to nonneuronal cells and likely also between different neuronal subtypes, which could influence epigenetics on the HSV genome. In addition, expression of distinct noncoding RNAs in neurons could modulate recruitment or activity Polycomb group proteins on viral genomes. There is evidence for of neuronal-specific long noncoding RNAs that can interact with Polycomb proteins and regulate gene expression important in neuronal identify, function, and plasticity [18]. The neuronal-specific HSV LAT also regulates the association of heterochromatin on the HSV genome in latency, and there is evidence that the LAT alters the levels of H3K27me3 and H3K9me2/3 on the latent genome [4,6,7]. The mechanisms by which the LAT regulates the HSV epigenome remain to be determined. Despite evidence that the LAT does not directly recruit the repressive PRC2 complex [9], at least in samples studied from the whole trigeminal ganglia during latency establishment, it is still possible that the LAT modulates deposition or maintenance of particular forms of heterochromatin. Further investigation analyzing different subtypes of heterochromatin or individual viral genomes will likely be required to determine any nuanced effects of the LAT on the viral epigenetic structure. Finally, the HSV genome may have a distinct higher-order chromatin structure in neurons that could regulate heterochromatin deposition. The chromatin insulator protein, CTCF, stabilizes three-dimensional DNA interactions to form domains containing genes in similar transcriptional states [19]. CTCF binding sites on the viral genome appear to have differential effects on viral gene expression in neurons, and there is evidence that CTCF-binding can modulate H3K27me3 deposition on the latent genome [20]. Given the potential for CTCF to regulate the epigenetic structure of the latent genome, more research is needed to determine how CTCF, as well as other epigenetic proteins, is recruited to viral genomes differently in neurons versus nonneuronal cells and how this affects the outcome of HSV neuronal infection.

## Is there a more reactivatable epigenetic structure?

HSV can reactivate in response to stimuli and reenter a lytic replication cycle. However, only a subpopulation will reactivate in response to a given stimuli [21,22], suggesting that latency is heterogenous and some viral genomes are more prone to reactivate. It is unclear if the marks found on the viral genome, such as H3K9me2/3 and H3K27me3, are enriched equally, if at all, on the same or different genomes. Beyond the heterogeneity in histone modifications, there are multiple reader proteins or protein complexes that can interact even with the same histone modification, further increasing the possible forms of epigenetic silencing on latent genomes. This is also supported by heterogeneity in viral genome localization to different subnuclear domains [23,24]. Therefore, viral genomes with different epigenetic structures and localized to different nuclear subdomains may be more or less prone to reactivate (Fig 1C). The heterogeneity in viral genome epigenetics may likely occur between different neurons, but could potentially occur on different genomes present in the same neuron. Viral genomes localized to one subnuclear domain; promyelocytic leukemia nuclear bodies (PML-NBs) are restricted for reactivation, at least in in vitro models of HSV latency [23]. Viral genome colocalization to PML-NBs only occurs in neurons exposed to type I interferon at the time of infection [23]. Although the downstream epigenetic changes to the HSV genomes that are associated with PML-NBs in neurons is not known, this indicates that exposure to different cytokines during initial infection can regulate the nature of latent infection and later ability to reactivate. It is likely that exposure to extracellular stimuli including cytokines, intrinsic differences in neurons themselves, and/or expression of viral factors (e.g., the LAT) regulate the nature of epigenetic silencing on the latent genome. Understanding whether there are subtypes of latent HSV epigenetic structures that are more or less able to undergo reactivation may enable to development of new therapies that prevent HSV reactivation occurring.

## What role does epigenetics have on the clinical outcomes of HSV infections?

Understanding how cellular epigenetic marks differ on latent HSV genomes between individuals could be important for understanding how HSV latency and reactivation can be heterogenous within a population and may result in more severe clinical phenotypes in certain individuals [25]. Numerous studies have linked HSV infection with the development of late onset Alzheimer disease, especially in individuals with the ApoE4 variant [25–27]. Recent evidence suggests that reactivation from latency occurs in waves [28,29], with the first wave being potentially reversible, raising the possibility of abortive reactivation events [30]. Whether abortively reactivated genomes revert back to the same epigenetic structure as their previous latent state remains to be determined. Abortive reactivation and consequential leaky lytic expression in the peripheral or central nerve systems may result in transient synthesis of viral lytic proteins that could impact neuronal function and could also induce a chronic inflammation, which could have significant impacts on the health of the nervous system (Fig 1D). Therefore, with upwards of 50% of the aging population having HSV infection, understanding the epigenetics surrounding HSV latency could have direct impacts on our understanding of clinical outcomes of HSV infection and potentially result in the development of new therapeutics to prevent even abortive reactivation events.
